# A case of polymicrogyria in macaque monkey: impact on anatomy and function of the motor system

**DOI:** 10.1186/1471-2202-10-155

**Published:** 2009-12-23

**Authors:** Eric Schmidlin, Christophe Jouffrais, Patrick Freund, Patrizia Wannier-Morino, Marie-Laure Beaud, Eric M Rouiller, Thierry Wannier

**Affiliations:** 1 Unit of Physiology and Program in Neurosciences, Department of Medicine, Faculty of Sciences, University of Fribourg, Chemin du Musée 5, CH-1700 Fribourg, Switzerland; 2 IRIT, Université de Toulouse and CNRS, 133 route de Narbonne, 31062 Toulouse cedex 9, France

## Abstract

**Background:**

Polymicrogyria is a malformation of the cerebral cortex often resulting in epilepsy or mental retardation. It remains unclear whether this pathology affects the structure and function of the corticospinal (CS) system. The anatomy and histology of the brain of one macaque monkey exhibiting a spontaneous polymicrogyria (PMG monkey) were examined and compared to the brain of normal monkeys. The CS tract was labelled by injecting a neuronal tracer (BDA) unilaterally in a region where low intensity electrical microstimulation elicited contralateral hand movements (presumably the primary motor cortex in the PMG monkey).

**Results:**

The examination of the brain showed a large number of microgyri at macro- and microscopic levels, covering mainly the frontoparietal regions. The layered cortical organization was locally disrupted and the number of SMI-32 stained pyramidal neurons in the cortical layer III of the presumed motor cortex was reduced. We compared the distribution of labelled CS axons in the PMG monkey at spinal cervical level C5. The cumulated length of CS axon arbors in the spinal grey matter was not significantly different in the PMG monkey. In the red nucleus, numerous neurons presented large vesicles. We also assessed its motor performances by comparing its capacity to execute a complex reach and grasp behavioral task. The PMG monkey exhibited an increase of reaction time without any modification of other motor parameters, an observation in line with a normal CS tract organisation.

**Conclusion:**

In spite of substantial cortical malformations in the frontal and parietal lobes, the PMG monkey exhibits surprisingly normal structure and function of the corticospinal system.

## Background

Polymicrogyria is a developmental malformation of the cerebral cortex, characterized by multiple small gyri with abnormal cortical lamination [1]. PMG can be unilateral or bilateral and its extent varies from focal PMG in otherwise normal brain to diffuse PMG with multiple other brain abnormalities. The spectrum of clinical manifestations ranges from normal individuals, with only selective impairment of cognitive function [2] and no or easily controlled epilepsy, to patients with severe encephalopathy and intractable epilepsy [3]. Motor and cognitive deficits such as a delay in development [4], or congenital contractures [5] are commonly described in patients suffering from PMG. Microscopically, two histological types of PMG were recognized: a simplified four layered form and an unlayered form [6]. The two types of PMG may coexist in contiguous cortical areas [7]. Recent report provides evidence that PMG areas are functional [8].

The present report describes a case of spontaneously occurring PMG in a macaque monkey for which tracing of corticospinal projections had been obtained. Moreover, the animal was involved in a study on the mechanisms of bimanual coordination, and its PMG was discovered after sacrifice. The first goal of the present report was to present in more details the general morphological traits of the PMG brain. More specifically, we sought to establish which brain regions and how the laminar pattern of the cerebral cortex have been affected by the PMG. In human patients with a unilateral PMG, the CS tract originating from the affected hemisphere presented an altered structure in DTI and fMRI investigations [9]. The second aim of the present case report in monkeys was to evaluate whether the cortical malformations affected the characteristics of the corticospinal projections. For this purpose, the anterograde tracer Biotinylated Dextran Amine (BDA) was injected unilaterally in the electrophysiologically identified hand representation of the presumed primary motor cortex. Finally, the motor capacity of the PGM macaque was compared with that of a normal macaque monkey, both trained to perform the same motor task, namely a modified version of the so-called "reach and grasp drawer" task [10].

## Results

The PMG monkey described in this study is the only case of cortical malformation ever observed in our laboratory.

### 1) Cortical structure

**Figure 1:**
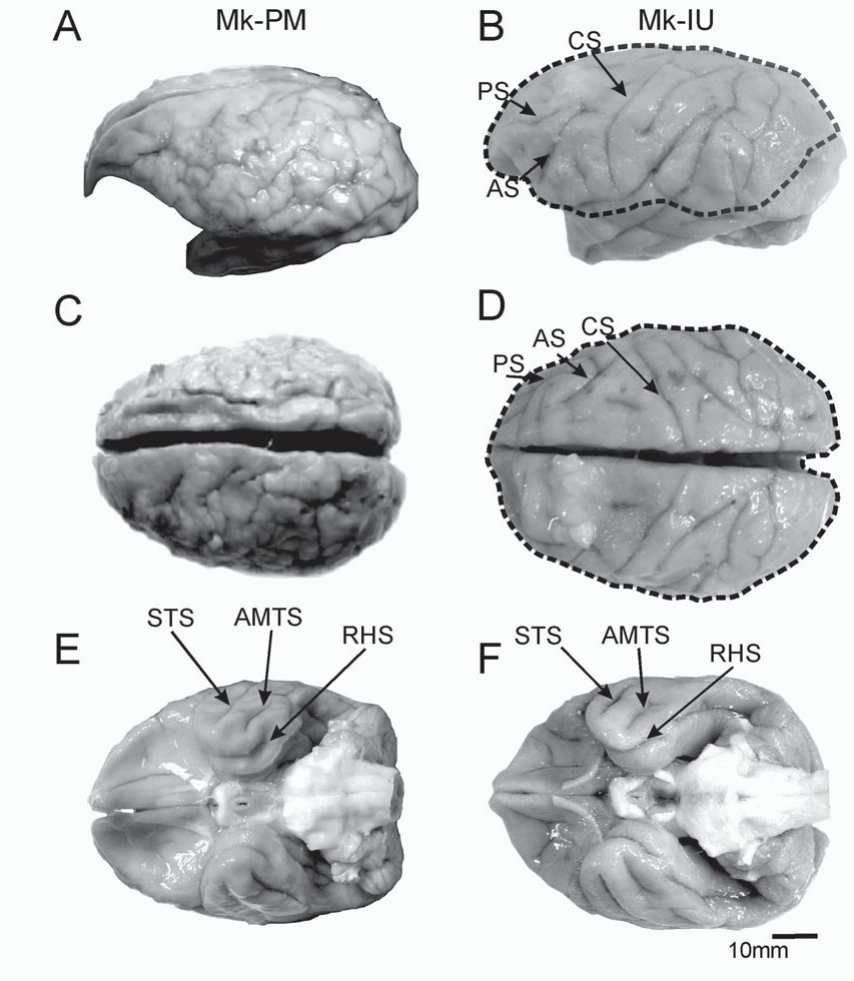
**macroscopic views of the brain**. Macroscopic appearance of the brain of the PMG monkey Mk-PM (A, C, E) and of a normal monkey Mk-IU (B, D, F) in lateral (A and B), dorsal (C and D) and ventral (E and F) views. Comparison of panels A and C with B and D shows a clear excessive number of small gyri in Mk-PM, and a loss of the normal topography of the brain, such as the disappearance of the normally well defined arcuate (AS), central (CS) or principal (PS) sulci. Nevertheless, the topographical organization of the ventral part of the PMG brain (E) is closer to that of the normal brain (F), as the superior temporal (STS), the anterior middle temporal (AMTS) or the rhinal sulci (RHS) are clearly identifiable. The extent of the cortical malformation in Mk-PM was projected over the healthy brain of Mk-IU (dashed line).

The topographical analysis of the PMG monkey brain showed an abundance of small gyri, affecting mainly the frontal and parietal lobes. In the PMG monkey, the structure of the sulci were hardly identifiable on both hemispheres (Fig. 1A and 1C), and did not display the arrangement normally observed (Fig. 1B and 1D). In contrast, the ventral aspects of the brain, particularly the occipital and the temporal lobes appeared macroscopically normal (Fig. 1E), with individual sulci exhibiting a pattern closely resembling that observed in normal monkeys (Fig. 1F). To tentatively represent the extent of cortical malformation, the border of the affected territories were superimposed on the healthy brain of Mk-IU in panels B and D (dashed line).

**Figure 2:**
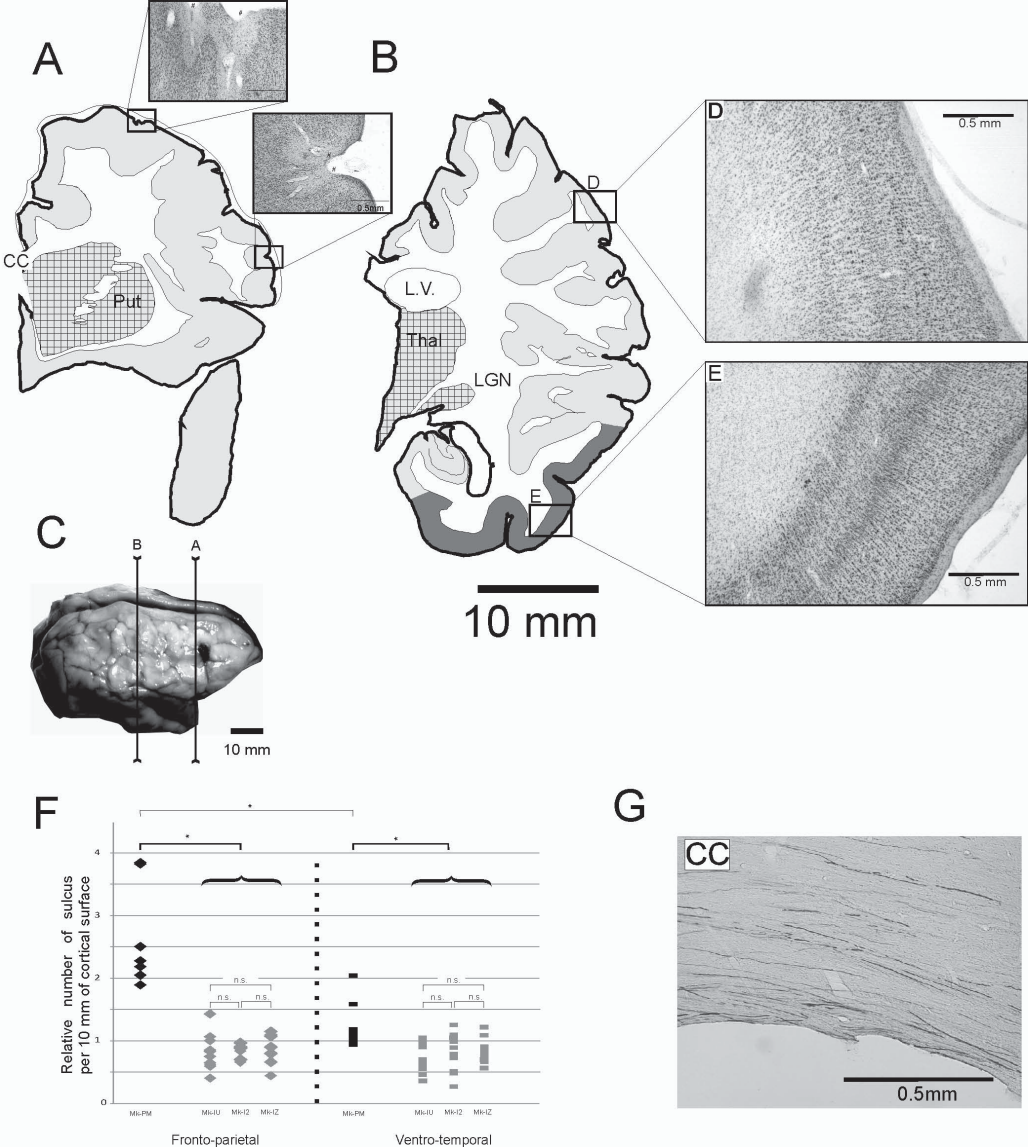
**Reconstructions of frontal Nissl-stained sections in PMG monkey**. **Panels A and B: **Location of cortical malformation on coronal sections in the right hemisphere at two rostrocaudal levels. Sub-cortical structures, such as the thalamus (Thal.), the lateral geniculate nucleus (LGN), the putamen (Put) or the corpus callosum (CC) are visible and they do not present conspicuous abnormalities, but the lateral ventricle (L.V.) is enlarged, including two enlarged photo micrograph of the cortex showing the microscopic cortical ectopic sulci (#), where the layer I is clearly distinguishable. Light grey: unlayered cortex. Dark grey: normally layered cortex: Squared: subcortical nuclei **Panel C: **Photograph of the cerebral cortex in Mk-PM with the position of the sections depicted in panels A and B. **Panels D and E: **The cortical organization in layers is lost in the parietal lobe (D) but normal in the inferior temporal lobe (E). **Panel F: **Diagram showing the distribution of the number of sulci per cortical length (number of sulcus per 10 mm) in the PMG monkey (black) and in the three control monkeys, Mk-IU, Mk-I2 and Mk-IZ (grey). Diamonds represent the results obtained in the frontoparietal region and bars in the ventro-temporal region. N.s is for non-statistically significant. **Panel G: **Photomicrograph of the Corpus Callosum (CC) in the right hemisphere of Mk-PM showing large quantity of BDA stained fibers. Scale bar: 500 microns.

**Figure 3:**
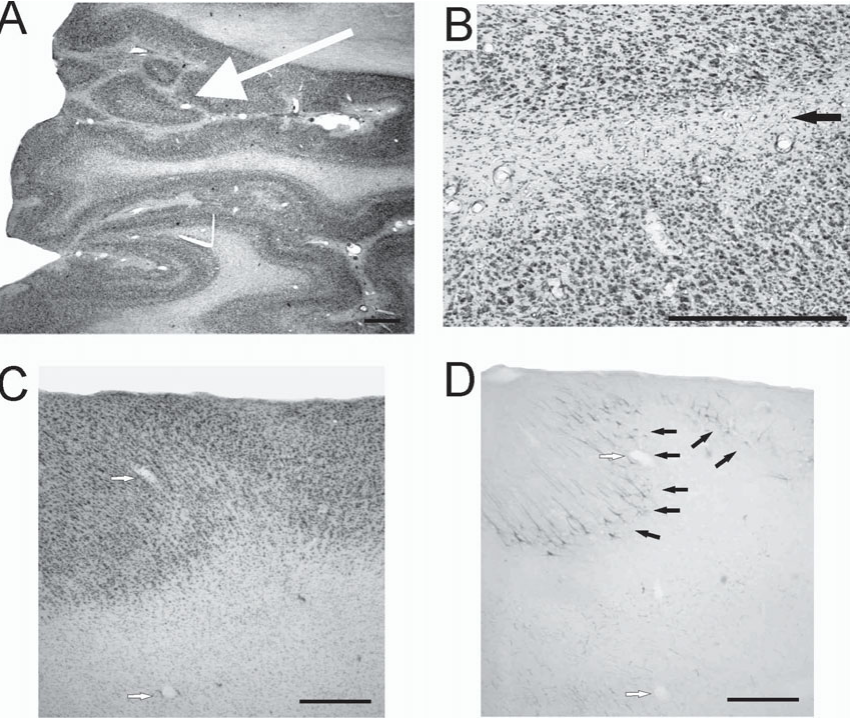
**Abnormalities in cortical organization**. **Panel A: **Photomicrograph of a Nissl stained section demonstrating the disorganization of the microgyrial cortex. The layered organization is irregular and anomalous, with the formation of small islets (arrow). **Panel B: **Photomicrograph showing the lack of lumen and of pia between two facing portions of cortex in a sulcus, resulting in the fusion of both layers I in a unique strip of white matter (black arrow). **Panel C: **Photomicrograph of a Nissl stained section showing the absence of well defined laminar organization. **Panel D: **Photomicrograph of a SMI-32 stained section adjacent to the section presented in panel C. Note that the SMI-32 positive neurons occupy positions in the cortex which vary strongly with regard of its distance to the cortex surface (black arrows). White arrows in panels C and D indicate the same blood vessels. Scale bars: 500 microns.

The cortical surface of the brain in the frontal and parietal lobes presented a disorganized aspect with numerous small gyri (Fig. 2A, B) delimited by intermingled invaginations (Fig. 3A arrow). In large regions of the frontal, parietal and temporal lobes, only layer I was clearly identifiable in Nissl stained sections, the remaining cortex failing to present a well characterized laminar organization (Fig. 2D). In ventral regions, a normal laminar organization in 6 layers was preserved (Fig. 2E). In some sulci, the cortical surface was not covered with a pial lamella, and the layers I of the two facing banks were fused (Fig. 3B). Locally, the normal laminar organization was disrupted in Nissl stained sections (Fig. 3C) as well as in SMI-32 stained sections (Fig. 3D). Indeed, in the PMG monkey, the SMI-32 positive neurons were often located at a distance from the surface that varied over a short rostro-caudal interval, and were not always oriented perpendicularly to the brain surface (Fig. 3D).

**Figure 4:**
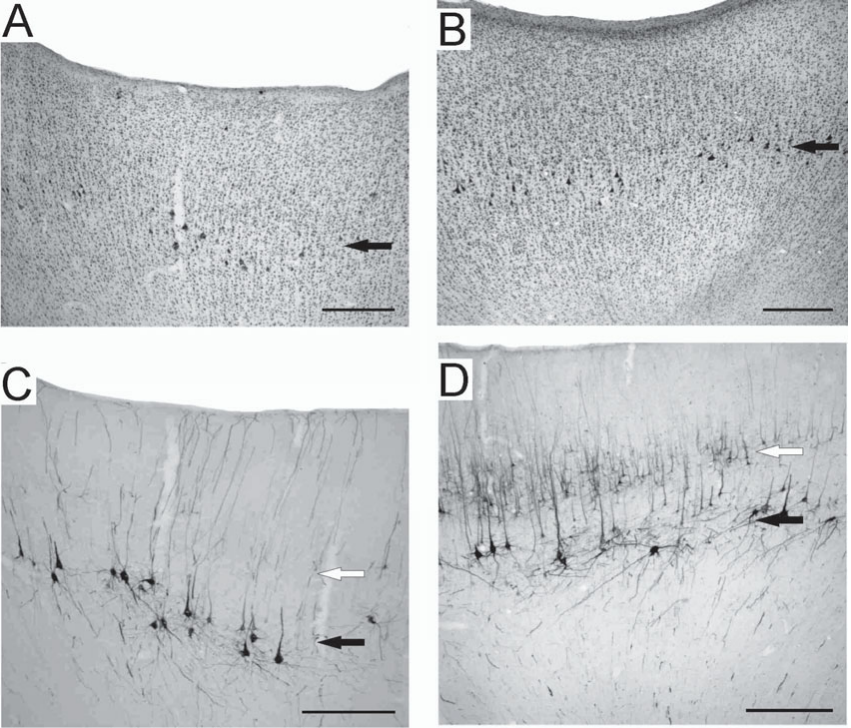
**Cytoarchitecture of the micro-excitable cortex**. Photomicrographs of motor cortex of the right hemisphere in the PMG monkey (A: Nissl staining; C: SMI-32 staining) and in the normal monkey (B: Nissl staining; D: SMI-32 staining). Black arrows show the location of pyramidal cells in layer V, whereas the open arrows show the location of layer III. Note the absence of layer III SMI-32 stained neurons in Mk-PM. Scale bars: 500 microns.

In the cortical region where ICMS elicited movements of the hand and where the laminar organization was identifiable, large pyramidal neurons were observed in Nissl and in SMI-32 stained material (Fig. 4A and 4C, black arrows) at a depth corresponding to layer V in normal monkeys (Fig. 4B and 4D, black arrows). However, in normal monkeys, numerous layer III pyramidal neurons were also SMI-32 positive (Fig. 4D, white arrow), whereas only few were recognizable in the PMG monkey (Fig. 4C).

When compared to normal monkeys (Mk-IU, Mk-I2 and Mk-IZ), the frequency of sulci measured on coronal sections in the fronto-parietal as well as in the ventro-temporal cortical regions was significantly higher in the PMG monkey (p < 0.05, Mann and Whitney with Bonferroni correction for multiple comparisons). Furthermore, in the PMG monkey, the frequency of sulci in the fronto-parietal cortex was nearly twice as high than that of the ventro-temporal region (Fig. 2F; p < 0.05, Mann-Whitney). No statistically significant difference was observed in the three normal monkeys. These differences do not reflect changes in the volume of the cortex, as at a comparable rostro-caudal position, the measured distance between the corpus callosum and the external part of the lateral fissure is similar among all animals.

In contrast to the few SMI-32 positive neurons detected in layer III of the presumed M1 area of the PMG-monkey, injections of BDA in this cortical region in the left hemisphere stained a large number of fibers in the corpus callosum (Fig. 2G) and several retrogradely labelled neurons were found in the right hemisphere in the frontal lobe. This observation suggests that a significant number of pyramidal neurons in lamina III are present, but do not express the neurofilament recognized by the SMI-32 antibody. As the cortical structure of the PMG brain is also disturbed in the contralateral side, it was not possible to assess the exact areas where projections were terminating and where callosal neurons were stained.

**Figure 5:**
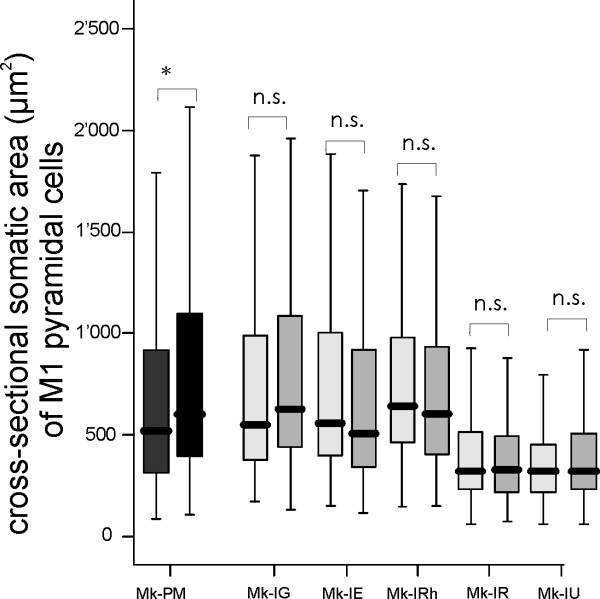
**Somatic cross-sectional areas of SMI-32 positive neurons in layer V in motor cortex**. Box and whisker plots showing the distribution of somatic cross-sectional areas of SMI-32 positive neurons in layer V in motor cortex for the PMG monkey (dark grey) and four normal monkeys (light grey). In the box and whisker plots, the thick horizontal line in the box corresponds to the median value, whereas the top and bottom of the box are for the 75 and 25 percentile values respectively. The top and bottom extremities of the vertical lines on each side of the box are for the 90 and 10 percentile values, respectively. Mk-PM exhibited a significant inter-hemispheric difference of cross-sectional soma area (*p < 0.0001; Mann and Whitney test) whereas, in the normal monkeys, the difference was not statistically significant (n.s. p > 0.05; Mann and Whitney test). For each monkey, the left box and whisker plot corresponds to data from the left hemisphere.

The analysis of the cross-sectional area of SMI-32 positive pyramidal cells in the putative layer V of M1 showed a statistically significant difference of somatic size between the left and the right hemispheres of the PMG monkey, a difference that was not observed in normal monkeys (Fig. 5). However, the somatic size of SMI-32 stained pyramidal cells in the PMG monkey is comprised in the range found in normal monkeys. As M1 is not well defined in the PMG monkey, it is difficult to ascertain that the measures were done on neurons placed in equivalent regions. The relative number of SMI-32 positive cells in layer V was not different among all monkeys.

### 2) The corticospinal (CS) projection

#### 2a) Crossed versus uncrossed CS projections

**Figure 6:**
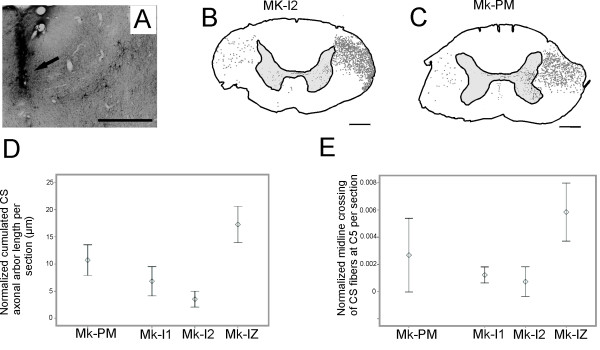
**Corticospinal projections in PMG monkey**. **Panel A: **Site of BDA injection in M1 hand area of the left hemisphere (arrow), close to identified layer V pyramidal neurons. **Panels B and C: **Reconstructions of BDA stained corticospinal (CS) fibers in coronal sections of the cervical spinal cord at the C5 level, as a result of BDA injection in the right motor cortex of a normal monkey (B) and in the left motor cortex of the PMG monkey (C); scale bar 1 mm. For better visual comparison of both reconstructions, the reconstruction in panel C was drawn with the left spinal side on the right side of the drawing. Grey dots indicate the location and distribution of the CS axons in the white matter. In both monkeys, most fibers were found in the dorsolateral funiculus (DLF) contralateral to the injection site, the rest running along the dorsolateral and ventral funiculi ipsilateral to the injection site. In comparison to normal monkeys, slightly fewer CS fibers were found in the grey matter ipsilateral to the injection side in the PMG monkey. **Panel D: **Normalized cumulated axonal arbor length of corticospinal projections in the cervical grey matter in the PMG monkey (black) and in three normal monkeys (grey). **Panel E: **Number of midline crossing CS fibers at cervical level C5. The number of fibers crossing the midline was normalized by dividing it by the total number of labelled CS fibers present in the white matter (see methods for detail).

In normal macaque monkeys, after a unilateral BDA injection in M1, 85-95% of the CS fibers were found in the opposite dorsolateral funiculus whereas 5-15% were in the ipsilateral dorsolateral and ventral funiculi (Fig. 6B). In the PMG monkey, BDA injections were placed in a cortical territory where ICMS elicited contralateral hand movements (Fig. 6A). This territory was later found to contain large pyramidal neurons. The proportions of BDA labelled CS axons in the contralateral and ipsilateral cervical white matter of the PMG monkey were 95% and 5%, respectively (Fig. 6C), thus comprised within the range found in normal monkeys.

#### 2b) Density of CS axonal arbors in the grey matter

The macroscopic structure and general histology of the spinal cord of the PMG monkey was normal. When compared to three normal monkeys, the PMG monkey exhibited a comparable CS arborization density in the grey matter at C5 (Fig. 6D; Table 1). The projection pattern of CS axonal arbors within the grey matter did not differ with respect to density nor to spatial distribution, as the CS arbors in the PMG monkey terminated mainly within the intermediate zone (Rexed laminae IV-VII), contralateral to the side of BDA injection (Fig. 6B, C), as in normal monkeys.

**Table 1:** Quantitative anatomical data for the CS tract tracing.

Monkey	Mk-PM PMG	Mk-I1 Intact	Mk-I2 Intact	Mk-IZ Intact
Volume of BDA Injected in M1 in μl	24	10	22.5	25.5

Number of BDA Injection sites	13	7	15	17

Survival time (in days) cumulated	41	21	45	51

Number of BDA-labelled CS axons at C5 in white matter	821	3133	1394	1884

% of uncrossed CS axons at C5	14.9	11.2	14.5	8.9

Normalized axon arbor length at C5 per section	10.71	6.88	3.58	17.32

Normalized number of axonal arbors crossing midline at C5	0.00268	0.00121	0.000717	0.00584

Survival time: number of days separating the injection of BDA in the contralesional M1 and the day of sacrifice of the animal.

#### 2c) CS axons crossing the midline at C5

We examined the same material to determine whether BDA-labelled CS axon collaterals crossed the midline in the grey matter (see methods). The number of CS axons crossing the midline at C5 in each monkey was normalized to the total number of labelled CS axons, in the white matter. On average, the PMG monkey exhibited a higher number of midline crossing CS axons, comprised within the range found in normal monkeys (Fig. 6E).

### 3) Magnocellular part of the red nucleus (RNm)

**Figure 7:**
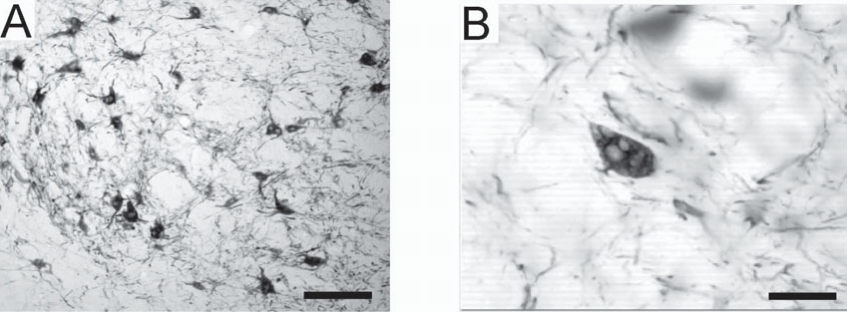
**Abnormal vesicles in RNm neurons**. SMI-32 staining of the red nucleus pars magnocellularis (RNm) in the PMG monkey. The general structure of the nucleus is comparable to that observed in normal monkeys (A), but abnormal accumulation of vesicles were observed in SMI-32 positive RNm neurons (B). Scale bars: (A) 200 μm; (B) 50 μm.

In the RNm of the PMG monkey, numerous neurons presented large vesicles (Fig. 7), a histological feature not observed in normal monkeys. We also investigated whether the number and somatic size of SMI-32 positive RNm neurons differed in the PMG monkey. The mean number per section and somatic size of SMI-32 stained RNm neurons was 131 16.1 μm^2 ^(+- SD) +- 11.5. These values are in the ranges obtained in normal monkeys (Mk-IR: n = 174, 19.3 +- 9.7; Mk-IE: n = 114, 12.8 +- 9.9; Mk-IZ: n = 93, 13.5 +- 7.8; Mk-IRh: n = 120, 14.7 +- 9.7).

### 4) Intracortical microstimulation and manual dexterity data

During the two years of behavioral training and single neuron recording, no deficit in learning and executing the demanding reach and grasp drawer task was detected in the PMG monkey, the pathology being discovered post-mortem.

**Figure 8:**
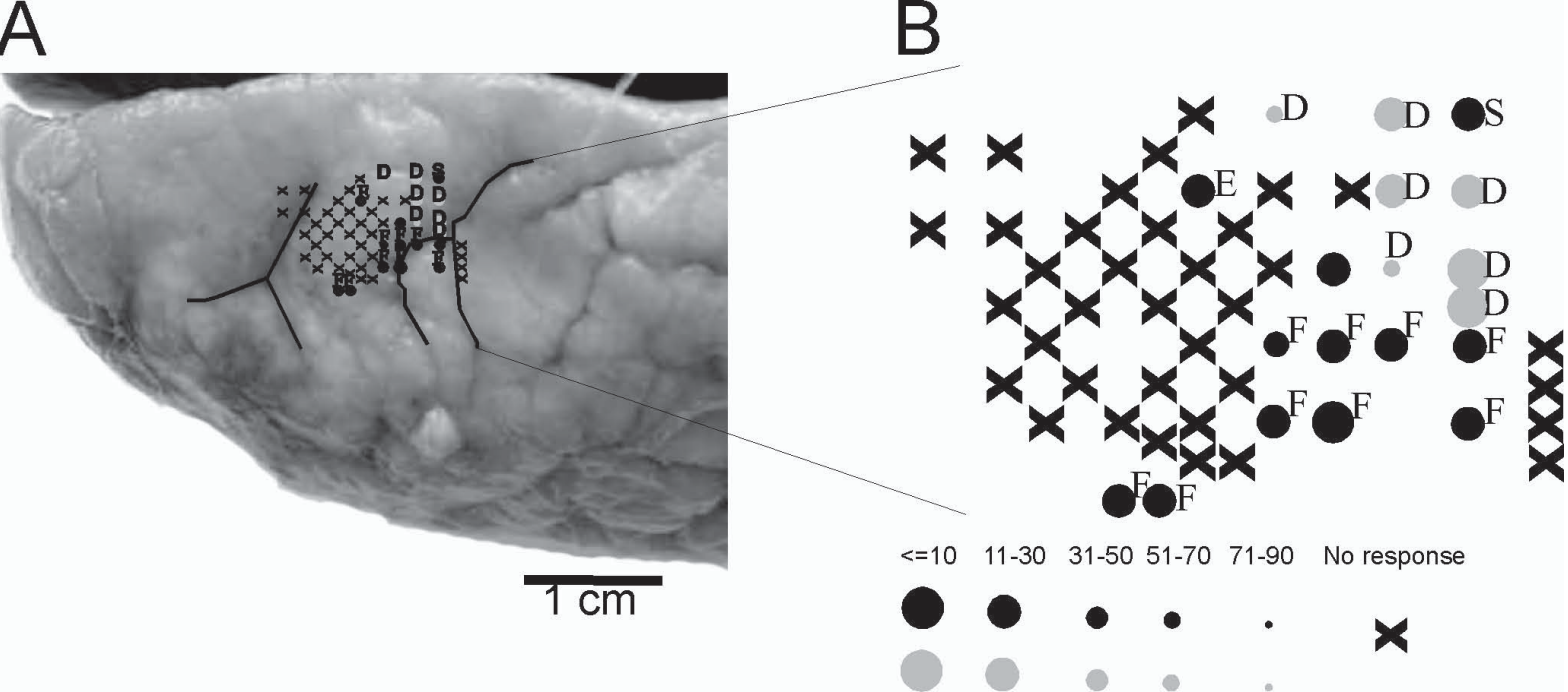
**Functional organisation of M1 in the PMG monkey**. **Panel A: **Left frontal lobe region of the brain of the PMG monkey with the map of the sites where intracortical microstimulation (ICMS) has been performed at a stereotaxic position corresponding to M1 in normal monkeys. Note the difficulty to identify the central sulcus and the precentral gyrus. **Panel B: **Map of the sites where ICMS has been performed, with indications on the body part of the observed movements and of the minimal current required to elicit the movement. D: digit, F: face, E: elbow and S: shoulder. X: No response at 80 microamps of stimulation intensity.

Intracortical microstimulation (ICMS) was conducted in the PMG monkey to confirm that single unit recordings had been obtained from the hand area of M1. The general organization of this region in the PMG monkey corresponded to that of normal monkeys: ICMS along tracks located laterally to the hand area elicited contractions of facial muscles whereas proximal movements (shoulder muscles) were elicited along tracks located medially to the hand area (Fig. 8). The ICMS thresholds to elicit joint movements were in the range observed in normal monkeys, typically at current intensities smaller than 10 microamps (Fig. 8B).

The extracellular single neuron activities recorded in motor areas of the left hemisphere while the PMG monkey performed the reach and grasp drawer task were similar to those obtained in normal monkeys. Epileptic seizures were never observed.

**Figure 9:**
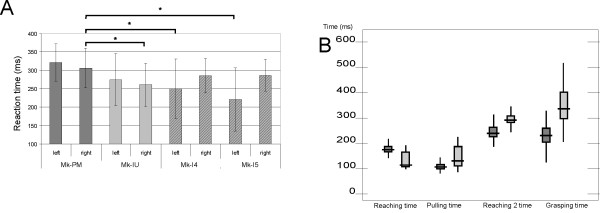
**Behavior: reach and grasp drawer task**. **Panel A: **Reaction times (RT) for each hands in the behavioral unimanual tasks for the 4 monkeys involved in the behavioral reach and grasp drawer task. In each case, the hand with which the PMG monkey (Mk-PM) exhibited the shortest average RTs (right) has been statistically tested against the fastest hand of the 3 normal monkeys (Mk-IU, Mk-I4, Mk-I5) using a Mann-Whitney test. **Panel B: **Movement times corresponding to the following movement components: First reaching time: from 'hand movement onset' to 'touch knob', Pulling time: from 'touch knob' to 'drawer fully open', Second reaching time: from 'grasping hand movement onset' to 'enter drawer's well', Grasping time: from 'hand penetrating in the well' to 'hand out of the well'.

After 7 to 10 months of training, the PMG monkey reached a success rate of 90 to 95% in the reach and grasp drawer task, a performance similar to that of the normal monkey (MK-IU). On the other hand, the reaction times (RTs) were significantly longer in the PMG monkey to perform the unimanual task with the fastest hand, as compared to the normal monkey (Mk-IU) (Fig. 9A Student's t-test, p < 0.0001). The PMG monkey also showed significantly longer RTs with the fastest hand as compared to two other monkeys (Mk-I4 and Mk-I5) involved in a former version of the reach and grasp drawer behavioral task, as reported previously [11] (Fig. 9A). The longer RT is not associated to motor impairment as in three out of four measures of motor capacity in the behavioral task (reaching times, pulling time, grasping time; Fig. 9B), the PMG monkey was significantly faster than the intact monkey (Mk-IU).

## Discussion

The present case of brain malformation in a macaque monkey can be diagnosed as a PMG according to previously reported criteria, namely disorganized cortical gyri in excessive number in both hemispheres and local loss of laminar organization in the frontal and parietal cortices [4]. According to the extent of the cortical malformation, the PMG corresponds to a bilateral frontoparietal polymicrogyria (BFPP), sparing most parts of the occipital and the temporal lobes, at both macro- and microscopic levels.

However, in the PMG monkey, the cortical region stereotaxically corresponding to the motor cortex of normal monkey contained large pyramidal neurons in layer V, giving rise to corticospinal projections. Moreover, ICMS in this region elicited movements at current thresholds similar to those reported for the motor cortex of normal monkeys [12,13].

In contrast to normal monkeys, only few SMI-32 positive layer III pyramidal neurons were present in the motor cortex of the PMG monkey. Despite the loss of SMI-32 staining in layer III in the motor cortical region, interhemispheric connections were seen between the left hemisphere (BDA- injections) and the right hemisphere (BDA labelled fibers in the Corpus Callosum and labelled neurons in the frontal lobe). As interhemispheric projections originate mostly from layer III pyramidal neurons, their presence in Mk-PM indicates that the loss of SMI-32 staining rather corresponds to a phenotypical change of layer III pyramidal neurons than to an absence of such cells.

The small but significant difference in soma size of layer V SMI-32 positive neurons observed between both hemispheres could result from observations made in somatotopically different regions. Due to the cortical disorganization in Mk-PM, it is difficult to ascertain that two regions equivalently located on both hemispheres project to the same level of the spinal cord [14].

After BDA injections in the ICMS identified hand area in M1, a large number of BDA stained axons were observed in the white matter at cervical levels. Their distribution and density were similar to that of normal monkeys [15], indicating that the basic structure of the corticospinal tract was preserved in the PMG monkey. This observation contrasts with recent imaging reports showing a decreased density of CS projections in patients suffering from PMG affecting only one hemisphere [9,16]. PMG is essentially a cortical malformation. Macroscopically, the subcortical structures appear entirely normal (Fig. 2). However, one cannot exclude some minor, microscopical changes in subcortical structures, as indeed found with the large number of vesicles observed in RNm neurones. The soma size of SMI-32 positive neurons in the RNm of the PMG monkey was however in the same range of that reported for normal monkeys [17]. As no motor impairment was observed, we have no direct indication that the histological changes that we observed in some RNm neurons altered the function of the red nucleus.

The absence of epileptic episodes in the PMG monkey contrasts with the study of Chang and colleagues [4] where all patients showed epileptic seizures or cognitive delays at different levels of severity, but is in line with the study of Teixeira and colleagues [18], where a majority of 40 patients diagnosed as suffering from PMG presented normal EEG recordings. The difference observed between these two studies in human subjects may be due to the heterogeneity of the localization of the cortical malformation.

The present study reports on a PMG monkey engaged in a sophisticated conditional delayed motor task (requiring 6-12 months training), comprising four conditional behavioral responses instructed by 4 visual cues, with a direct comparison to a normal monkey engaged simultaneously in the same behavioral task. The most striking observation is that the PMG monkey, in spite of considerable malformation of the cerebral cortex (Figs. 1, 2, 3, 4), performs apparently as well as the normal monkey, both in terms of training curve and stabilized motor performance after training. However, the behavioral data derived from the reach and grasp drawer task showed that the PMG monkey (Mk-PM) had significantly longer RTs than the normal monkeys (Fig. 9A). Nevertheless, after initiation of the movement sequence, the time intervals between different movement components of the overall motor response did not show any systematic variation (longer or shorter). Indeed, the first reaching time (interval between movement onset and drawer knob grasping) in the unimanual task was longer in the PMG monkey but the pulling time (opening of the drawer), the second reaching time and the grasping time were slightly shorter (Fig. 9B). This observation of "normal" motor control in the PMG monkey is in line with a generally normal organization of its corticospinal tract (Fig. 6). Furthermore, "normal" motor control in the PMG monkey is also consistent with the electrophysiological data, namely the presence of low (normal) threshold ICMS effects observed in the presumed hand area of the primary motor cortex (Fig. 8). The latter ICMS data are also coherent with a normal density and appearance of large pyramidal neurons in layer V in the presumed motor cortex in the PMG monkey, as seen in SMI-32 staining (Fig. 4).

The significantly prolonged RTs in the PMG monkey may be associated to a deficit of attention. It has been shown that, in a delayed conditional task instructed with visual cue signals and requiring discrimination of a specific stimulus among irrelevant distracters, attention is under the control of top-down inputs from the lateral prefrontal cortex onto visual cortical areas [19]. The authors found a significant increase of RTs in the task after lesion of the lateral prefrontal cortex. In the present case, as the PMG involved the frontal lobe, the lateral prefrontal cortex may be affected, leading to a deficit of attention. Along this line, the disorganization of some cortical layers, and the decrease of the density of SMI-32 positive neurons in layer III (Fig. 4C), suggests that some cortico-cortical interactions may be abnormal in the PMG monkey.

## Conclusions

Overall, these data suggest that the PMG pathology may have affected some cortico-cortical connections (crucial for attention), but not the corticospinal projection as indicated by normal motor control in a well trained behavioral task.

## Methods

### Animals

The data were derived from eleven young adults (2-9 years old) macaque monkeys (Macaca mulatta or fascicularis, of either sex, weighing from 3.0 to 9.0 kg, see Table 2). Monkeys Mk-IG, Mk-IE, Mk-IRh, Mk-IR, and Mk-IZ were involved in previously published studies [17,20]. Surgical procedures and animal care were conducted in accordance with the Guide for the Care and Use of Laboratory Animals (ISBN 0-309-05377-3; 1996) and approved by local (Swiss) veterinary authorities. Details on the sacrifice of the animals at the end of the experiments and on histological processing are available in Additional file 1.

### Behavioral experiments

The PMG monkey (Mk-PM) and a normal monkey (Mk-IU) were enrolled in a conditional delayed bimanual dexterity task (see additional files 1 and 2 and additional Fig. S1), corresponding to a modified and more complex version of the so-called "reach and grasp drawer task" [10,21-23]. In order to locate the hand representation of the primary motor cortex (M1), an intracortical microstimulation (ICMS) mapping was performed, as described in detail earlier [13,24-28].

### Tracing experiments

In the PMG monkey and three normal monkeys, a craniotomy provided access to the cerebral cortex, allowing injections of the tracer BDA at physiologically defined loci in the M1 hand area of one hemisphere. Under propofol anaesthesia (Disoprivan, 3 mg/kg/h, i.v.), the craniotomy was performed in those four monkeys to expose the stereotaxic area corresponding to the motor cortex. In intact monkeys, injections of BDA were placed in the rostral bank of the central sulcus, following the central sulcus going from lateral (hand representation) to medial (leg representation). In the PMG monkey, the BDA injections were performed at side where ICMS elicited hand movements. Based on our previous experience of tracing the CS tract with BDA in monkeys, the survival time after BDA injection was set to three to four weeks [25].

### General morphology

To compare the frequency of sulci in the brain of the PMG monkey with that of normal monkeys, we counted the number of sulci in the frontoparietal and ventro-temporal lobes in the PMG monkey and in three control monkeys (Mk-IU, Mk-I2 and Mk-IZ), divided by the measured length of the corresponding cortical surface. The measurements were made on 5 coronal sections at 40× magnification, regularly distributed between the rostral end of the Nucleus Caudatus and the rostral end of the Lateral Geniculate Nucleus (LGN). On the rostral sections, where the lateral fissure is not present, the measure was obtained from the region delimited by the Corpus Callosum and the angle between the lateral and the ventral walls of the frontal lobe. A depression of the cortical surface was considered as a sulcus when we could identify a clear invagination of the layer I (Fig. 2A).

### Measurement of CS axonal arborization

At cervical level C5, in the grey matter, the presence of BDA labelled CS axonal arbors was investigated on five coronal sections at 400× magnification. Using Neurolucida^® ^software, each BDA-labelled axonal segment observed in the grey matter was traced and the cumulated axonal arbor length was then computed. As the number of BDA labelled CS axons varied across monkeys, the measures were normalized to the total number of BDA-labelled CS axons counted in the white matter at C5 level on three coronal sections (Table 2). Furthermore, on the five sections at C5 level, the numbers of CS fibers crossing the midline were counted, as previously reported [29]. For normalization, their cumulated number was divided by the total number of CS axons present at C5 level in the white matter.

**Table 2:** List of monkeys included in the present study with identification code.

	Mk-PM	Mk-I1	Mk-I2	Mk-IZ	Mk-IU	Mk-IR	Mk-IE	Mk-IRh	Mk-IG	Mk-I4	Mk-I5
	**PMG**	**Intact**	**Intact**	**Intact**	**Intact**	**Intact**	**Intact**	**Intact**	**Intact**	**Intact**	**Intact**

**Species**	**mul**.	**fasc**.	**fasc**.	**fasc**	**mul**.	**mul**.	**mul**.	**mul**.	**mul**.	**fasc**.	**fasc**

Age at sacrifice (years)	5	3.75	7.75	8	9	5	5	6.5	8	2	2.5

Study	BIM + RT + SF	SCI study	SCI study +SF	SCI study + RN + SF	Cortex BIM + RT + SF	Cortex + RN	Cortex+ RN	Cortex+ RN	Cortex	RT	RT

ICMS	Yes	No	No	No	Yes	No	No	No	No	Yes	Yes

Monkey Mk-PM exhibited a brain with PMG whereas the other 10 monkeys had a brain with normal appearance.

Under species, "mul." is for macaca mulatta whereas "fasc." is for macaca fascicularis.

Under ICMS (intracortical microstimulation), "Yes" refers to the four monkeys subjected to ICMS sessions in M1.

BIM + RT: Bimanual reach and grasp task study: assessment of motor performance in the present reach and grasp drawer task (see additional file 1 Fig. S1) with measures of RT's.

RT: Assessment of RT in a former version of the reach and grasp drawer task[11];

SF: Measurement of the Sulcus frequency in the cortex.

RN: Red Nucleus investigation study [17]; Cortex: Cortical anatomical investigation study [20];

SCI: Spinal cord injury study: monkeys used as control intact monkeys in previous studies on spinal cord injury, used here for comparison.

Numbers in brackets correspond to the reference number in the manuscript.

## Authors' contributions

ES sampled the behavioral data, performed the analysis of the BDA staining in the cervical cord, as well as the macroscopical analysis of the sulci in the brain. He also performed most of the statistical analysis of the data and part of the behavioral training. CJ was responsible for the behavioral training and electrophysiological recording in the monkeys as well as the analysis and the interpretation of the behavioral data. PF contributed to the BDA projections analysis wrote the first draft of the paper. PW carried out the morphological analysis of the cells and of their distribution in the Red Nucleus. MLB made the morphological analysis of the cortical neurons in the presumed motor cortex. EMR participated to the design and the coordination of the study, the interpretation of the data, the surgical, histological and behavioral procedures. TW participated to the design of the study, the analysis of the histological data, the coordination of the comments and the replies to the reviewers received from the co-authors. All authors read and approved the final manuscript.

## Supplementary Material

Additional file 1 **Figure depicting the behavioral task**. Schematic representation of the sequences required for executing the complex behavioral task. Click here for file

Additional file 2 **Additional methods**. Additional information about the procedures and legend of the additional file 1 figure S1. Click here for file
