# Supplementary figures and images for: A case of polymicrogyria in macaque monkey: impact on anatomy and function of the motor system
Source: BMC Neurosci. 2009 Dec 23;10:155. doi: 10.1186/1471-2202-10-155 (PMC2807873; doi:10.1186/1471-2202-10-155)

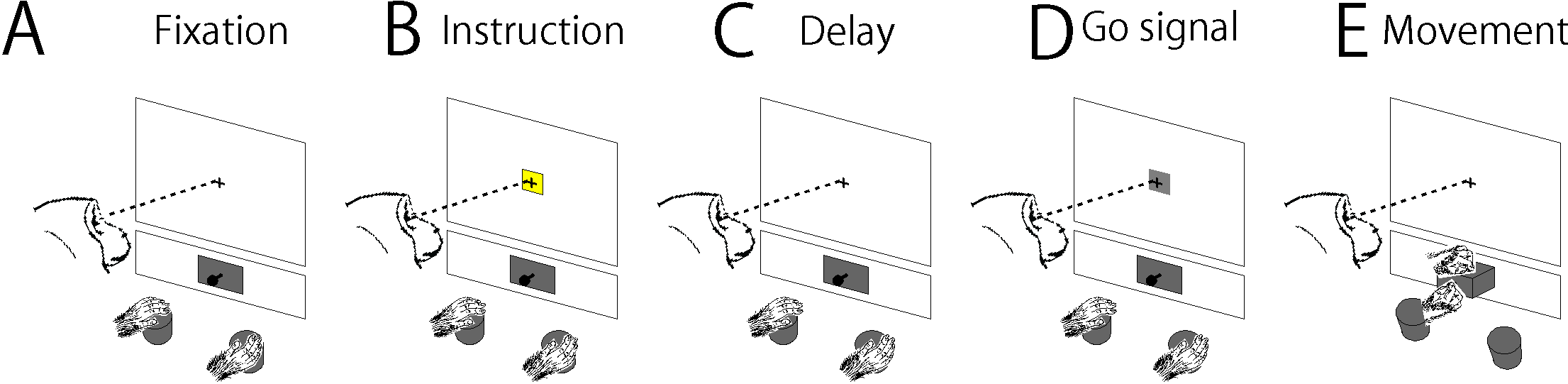

Supplement: Additional file 1 — Figure depicting the behavioral task. Schematic representation of the sequences required for executing the complex behavioral task. [file 1471-2202-10-155-S1.TIFF]
