# Supplementary material for: A case of polymicrogyria in macaque monkey: impact on anatomy and function of the motor system
Source: BMC Neurosci. 2009 Dec 23;10:155. doi: 10.1186/1471-2202-10-155 (PMC2807873; doi:10.1186/1471-2202-10-155)
Supplement: Additional file 2 — Additional methods. Additional information about the procedures and legend of the additional file 1 figure S1. [file 1471-2202-10-155-S2.DOC]

Additional file 2:

**Sacrifice**

At the end of each experimental protocol, the monkeys were sacrificed with a lethal dose of sodium pentobarbital (90 mg /kg i.p.). The transcardiac perfusion was initiated with saline (0.9%, 400 ml) followed by a 4% paraformaldehyde solution (3 liters; 0.1 M phosphate buffered; pH=7.6), a solution (2 l) of the same fixative containing 10% sucrose and ended with a 20% followed by a 30% sucrose solution (2 l each). The brain and spinal cord were dissected and placed in a 30% solution of sucrose in phosphate buffer for cryoprotection during few days. Frozen sections (50 µm thick) of the brain and of the spinal cord at the C5 level were cut in the frontal plane. BDA staining was revealed on one serie of spinal sections, as described in detail in previous reports [1, 2].

### **SMI-32 staining and histology**

The epitope recognized by the SMI-32 antibody lies on non-phosphorylated regions of the neurofilament protein and is only expressed by particular categories of neurons [3], [4]. In the healthy cerebral cortex, SMI-32 stains pyramidal neurons in layers III and V. In the red nucleus, SMI-32 labeled most neurons in the magnocellular part (RNm) [5]. For the analysis of the RNm, all sections containing SMI-32 stained RNm neurons were considered. In a region presumably corresponding to M1 (motor responses elicited by low intensity ICMS, presence of large pyramidal neurons and origin of corticospinal projections) and in the RNm, the number of SMI-32 positive neurons and their somatic cross-sectional area were measured at 400x magnification for each monkey on both right and left brain halves on 50 μm thick coronal sections, separated by 400 μm each. Statistical analysis of the data was then performed with the SPSS software for evaluating whether the cell number (Wilcoxon signed-rank, comparison left-right within the same animal) or their cross-sectional area (Mann and Whitney test) observed in the PMG animal differed from that of normal animals.

**Reach and grasp drawer task**

The modified reach and grasp drawer bimanual task consisted in reacting to the presentation of one out of four different visual signals by selecting one specific movement type out of a set of four possible appropriate movements to open a drawer containing a food reward. The movements consisted in opening a drawer with the left or the right hand and picking the food with the same hand (left or right unimanual movements), or in opening the drawer with either the left or the right hand and grasping the food reward with the other hand (left or right bimanual movements). The monkey initiated a trial by placing the two hands on sensitive start pads. After a random delay, one of four different visual signals (red, green, blue or yellow rectangles presented on a monitor facing the monkey) instructed the animal which one of the four different sub-tasks it had to perform **i)** **Unimanual Left Task:** pulling the drawer with the left hand, the drawer being blocked in the open position, and picking the reward placed inside the drawer using the same left hand (during the whole task, the right hand had to remain immobile on the corresponding start pad); **ii)** **Unimanual Right Task:** same as i) but using the right hand only; **iii)** **Bimanual Left Hand Pulling:** the monkey used first the left hand to pull and maintain open the drawer, following by grasping of the reward using the right hand; **iiii) Bimanual Right Hand Pulling:** same as iii), but inversion of the two hands, namely pulling with the right hand and grasping the reward with the left hand. In the four sub-tasks, after pulling, the drawer was automatically blocked in open position after pulling, but in the two bimanual sub-tasks, it was unblocked and closed by a spring if the monkey released the drawer handle. During the entire trial, the monkey had to maintain the gaze on a fixation point in the center of the monitor. The reaction time is the time interval between the go-signal and the onset of the reaching hand movement. The four sub-tasks were instructed to the monkey in a random sequence. As compared to previously published versions of the drawer task [6]; [7, 8]; [9], the present behavioral paradigm uses the same two unimanual sub-tasks whereas a unique bimanual sequence (chosen by the monkey) was replaced here by two imposed bimanual modes, instructed by a visual cue. In the two monkeys, after intensive training during about a year to reach a plateau of stable performance, a chronic recording chamber was implanted under deep anesthesia (see below), with bars to fixate the head (see e.g. [7] [9]), Single unit activity was then derived during daily sessions (about 2 hour’s duration) from the motor cortex while performing the task. These neurophysiological data will be reported in detail elsewhere.

Legend to supplementary figure

**Panel A:** Fixation: The monkey maintains his hands on the start pads and fixates the center point of the monitor.

**Panel B:** Instruction: Appearance at the center point of the monitor of the visual cue (colored square) determining the nature of the required behavioral task.

**Panel C:** Delay: Disappearance of the visual cue and delay period (1-2.5 sec.).

**Panel D:** Go signal: A grey square corresponding to the go signal appears at the centre point of the monitor.

**Panel E:** Movement: the animal starts the movement of the hand to perform the instructed bimanual or unimanual reach and grasp task.

1. Rouiller EM, Moret V, Tanne J, Boussaoud D: **Evidence for direct connections between the hand region of the supplementary motor area and cervical motoneurons in the macaque monkey**. *Eur J Neurosci* 1996, **8:**1055-1059.

2. Rouiller EM, Tanné J, Moret V, Kermadi I, Boussaoud D, Welker E: **Dual morphology and topography of the corticothalamic terminals originating from the primary, supplementary motor, and dorsal premotor cortical areas in macaque monkeys**. *J Comp Neurol* 1998, **396:**169-185.

3. Campbell MJ, Morrison JH: **Monoclonal antibody to neurofilament protein (SMI-32) labels a subpopulation of pyramidal neurons in the human and monkey neocortex**. *J Comp Neurol* 1989, **282:**191-205.

4. Tsang YM, Chiong F, Kuznetsov D, Kasarskis E, Geula C: **Motor neurons are rich in non-phosphorylated neurofilaments: cross-species comparison and alterations in ALS**. *Brain Res* 2000, **861:**45-58.

5. Wannier-Morino P., Schmidlin E., Freund P., Belhaj-Saïf A., Bloch J., Mir A., Schwab M.E., Rouiller E., Wannier T.: **Fate of rubrospinal neurons after unilateral section of the cervical spinal cord in adult macaque monkeys: effects of an antibody treatment neutralizing Nogo-A**. *Brain Res* 2008.

6. Kazennikov O, Hyland B, Corboz M, Babalian A, Rouiller EM, Wiesendanger M: **Neural activity of supplementary and primary motor areas in monkeys and its relation to bimanual and unimanual movement sequences**. *Neuroscience* 1999, **89:**661-674.

7. Kermadi I, Liu Y, Rouiller EM: **Do bimanual motor actions involve the dorsal premotor (PMd), cingulate (CMA) and posterior parietal (PPC) cortices? Comparison with primary and supplementary motor cortical areas**. *Somatosens Mot Res* 2000, **17:**255-271.

8. Kermadi I, Liu Y, Tempini A, Calciati E, Rouiller EM: **Neuronal activity in the primate supplementary motor area and the primary motor cortex in relation to spatio-temporal bimanual coordination**. *Somatosens Mot Res* 1998, **15:**287-308.

9. Wannier T, Liu J, Morel A, Jouffrais C, Rouiller EM: **Neuronal activity in primate striatum and pallidum related to bimanual motor actions**. *NeuroReport* 2002, **13:**143-147.
